# Portal vein thrombosis in liver cirrhosis: incidence, management, and outcome

**DOI:** 10.1186/s12876-017-0668-8

**Published:** 2017-10-25

**Authors:** Shunichiro Fujiyama, Satoshi Saitoh, Yusuke Kawamura, Hitomi Sezaki, Tetsuya Hosaka, Norio Akuta, Masahiro Kobayashi, Yoshiyuki Suzuki, Fumitaka Suzuki, Yasuji Arase, Kenji Ikeda, Hiromitsu Kumada

**Affiliations:** Department of Hepatology, Toranomon Hospital, Toranomon 2-2-2, Minato-ku, Tokyo, 105-8470 Japan

**Keywords:** Portal vein thrombosis, Danaparoid sodium, Liver cirrhosis

## Abstract

**Background:**

Portal vein thrombosis (PVT) is a serious complication in liver cirrhosis with portal hypertension. We examined the treatment, recurrence and prognosis of PVT in cirrhotic patients.

**Methods:**

The study subjects were all 90 cirrhotic patients with PVT treated with danaparoid sodium (DS) at our department between July 2007 and September 2016. The mean age was 68 years and mean Child-Pugh score was 7. All patients received 2500 U/day of DS for 2 weeks, and repeated in those who developed PVT recurrence after the initial therapy.

**Results:**

Complete response was noted in 49% (*n* = 44), partial response (shrinkage ≥70%) in 33% (*n* = 30), and no change (shrinkage <70%) in 18% (*n* = 16) of the patients after the initial course of treatment. DS treatment neither caused adverse events, particularly bleeding or thrombocytopenia, nor induced significant changes in serum albumin, total bilirubin, prothrombin time, and residual liver function. Re-treatment was required in 44 patients who showed PVT recurrence and 61% of these responded to the treatment. The cumulative recurrence rates at 1 and 2 posttreatment years were 26 and 30%, respectively. The recurrence rates were significantly lower in patients with acute type, compared to the chronic type (*p* = 0.0141). The cumulative survival rates at 1 and 3 years after treatment (including maintenance therapy with warfarin) were 83 and 60%, respectively, and were significantly higher in patients with acute type than chronic type (*p* = 0.0053).

**Conclusion:**

We can expect prognostic improvement of liver cirrhosis by warfarin following two-week DS therapy for the treatment of PVT in patients with liver cirrhosis safety and effectiveness. An early diagnosis of PVT along with the evaluation of the volume of PVT on CT and an early intervention would contribute to the higher efficacy of the treatment.

## Background

Liver cirrhosis represents the end stage of chronic diseases of the liver and is associated with life-threatening complications [1, 2]. The natural course of cirrhosis is largely affected by various pathologies, such as variceal bleeding, ascites, and infection [3, 4]. The major predictors of survival of patients with liver cirrhosis are Child-Pugh score; model for end-stage liver disease score; and various biochemical parameters, such as serum bilirubin, albumin, prothrombin time or international normalized ratio, creatinine, as well as encephalopathy and ascites [4, 5]. Recent evidence suggests the association between portal vein thrombosis (PVT) and survival of patients with liver cirrhosis [6], although the data are inconclusive.

Based on the increase in the use of technically-advanced noninvasive liver imaging modalities, PVT has been increasingly identified in patients with cirrhosis, with the estimated current prevalence of PVT in patients with cirrhosis of 0.6 to 26% [3, 7, 8].

The value of anticoagulation in the treatment of PVT in patients with cirrhosis remains controversial [9]. Cirrhosis is associated with bleeding diathesis based on the following factors: prolonged bleeding time, thrombocytopenia associated with hypersplenism, increased prothrombin time/international normalized ratio, reduced synthesis of coagulation factors, and secondary hyperfibrinolysis [10, 11]. More importantly, nearly half of patients with cirrhosis are diagnosed with gastroesophageal varices that can result in life-threatening bleeding events [12]. Taken together, these detrimental conditions greatly limit the use of anticoagulants for patients with cirrhosis. However, recent evidence indicates that both pro- and anticoagulation factors are concomitantly reduced in patients with cirrhosis [13], thereby maintaining a balance in the coagulation system [13–15]. Unfortunately, bleeding risk cannot be accurately assessed in patients with chronic liver disease by globally used coagulation tests [16, 17]. In addition, the occurrence of bleeding in patients with cirrhosis is not primarily dependent on hemostatic abnormalities, but on the severity of portal pressure, endothelial dysfunction, and bacterial infection [14]. Accordingly, the use of anticoagulants for patients with cirrhosis and PVT may be theoretically justified. However, convincing clinicians to prescribe anticoagulants to cirrhotic patients, especially those with decompensated cirrhosis, remains difficult.

Danaparoid sodium (DS) is a glycosaminoglucoronan derived from the same starting material, porcine intestinal mucosa, as unfractured heparin and low-molecular-weight heparins (LMWHs), but its extraction procedure excludes heparin and heparin fragments [18]. Danaparoid is a low-molecular-weight heparinoid consisting of heparin sulfate (84%), dermatan sulfate (12%), and chondroitin sulfate (4%). The mean mass of its components is approximately 6000 Da [18]. Its antithrombotic activity has been well established. Danaparoid catalyzes inactivation of factors Xa (FXa) and thrombin. Like most LMWHs, danaparoid exerts a stronger catalytic effect on inactivation of FXa by antithrombin (AT)-III, than on inactivation of thrombin by AT-III [19].

Anticoagulation therapy is definitely the most effective way to achieve recanalization of the portal vein, thereby improving the prognosis of patients with PVT. To our knowledge, the optimal management of PVT in individuals with cirrhosis has not yet been addressed by any consensus publication or practice guideline. In this study, we examined the response to treatment, recurrence rate and prognosis of PVT in a group of Japanese cirrhotic patients.

## Methods

### Patients

**Fig. 1:**
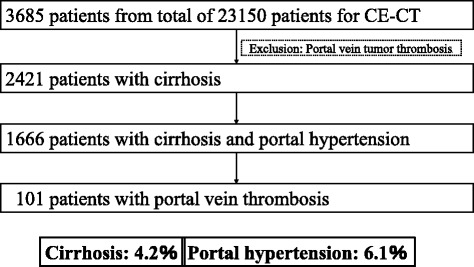
Incidence of portal vein thrombosis. Portal vein thrombus was recognized in 4.2% of cases of cirrhosis of the liver and 6.1% of cases of portal hypertension

In the past 9 years, 23,150 contrast-enhanced computed tomography (CECT) was performed at our hospital, among which 3685 were for patients with liver disease. Of these, 1264 patients had portal vein tumor thrombosis. Of the remaining 2421 patients with liver cirrhosis, 1666 patients were diagnosed with liver cirrhosis and portal hypertension. Among these, 101 patient had portal vein thrombosis. In other words, PVT was recognized in 4.2% of patients with cirrhosis of the liver and 6.1% of patients with portal hypertension (Fig. 1). Of the latter group, 90 patients were enrolled in the present study; they represented consecutive cirrhotic patients with PVT who were treated at our hospital with DS between July 2007 and September 2016. We divided the patients into two groups; 27 patients developed PVT within 1 month after hepatectomy (16 cases) or splenectomy (11 cases). We defined this group as an “Acute type”. Because CECT is often taken early postoperatively. And 63 patients developed PVT without particular cause. We defined this group as an “Chronic type”. Table 1 summarizes the clinical characteristics of the study patients as recorded before treatment, including patients of the two groups. For the entire group, the mean age was 68 years, 55% of the patients were males, 61 (72%) patients had hepatitis C virus-related cirrhosis, and the mean Child-Pugh score was 7. Hepatocellular carcinoma (HCC) without invasion of the bile duct, hepatic vein, or portal vein was diagnosed in 40 (47%) patients at the time of study enrolment.

**Table 1:** Clinical characteristics of cirrhotic patients with PVT

	Total	Acute type	Chronic type	*p*
Number of patients	90	27	63	
Age (years)^a^	68 (37–84)	62 (37–77)	70 (45–84)	0.0034
Male sex	47	16	31	0.1885
BMI (kg/cm^2^)^a^	23.0 (16.2–36.5)	23.9 (16.2–31.5)	22.9 (18.1–36.5)	0.1253
Etiology (hepatitis C viral infection)	61	19	42	0.3445
Clinical findings				
Hepatic encephalopathy (+)	5	2	3	0.7748
Ascites (+)	39	11	28	0.9955
Esophagogastric varices (+)	70	13	57	0.0000
HCC (+)	40	1	39	0.0000
Duration of DS treatment (14/28 days)	61/24	18/6	43/18	0.6042
Child-Pugh score	7 (5–12)	7 (5–10)	8 (5–12)	0.0143
Platelet count (μl)	8.0 (1.7–65.5)	20.0 (3.9–65.5)	7.1 (1.7–21.7)	0.0000
Serum albumin (g/dL)	3.1 (2.1–4.5)	3.1 (2.7–4.5)	3.2 (2.1–4.4)	0.5181
Total bilirubin (mg/dL)	1.3 (0.4–5.4)	0.9 (0.4–3.9)	1.5 (0.5–5.4)	0.0000
Aspartate aminotransferase (IU/L)	39 (15–177)	36 (17–103)	41 (15–177)	0.0427
Alanine aminotransferase (IU/L)	23 (0–119)	22 (11–78)	23 (0–119)	0.9222
NH_3_ (μg/dL)	70 (21–185)	44 (22–136)	78 (21–185)	0.0064
Prothrombin time (%)	68.3 (27.8–95.3	73.8 (50.0–85.6)	65.0 (27.8–95.3)	0.1010
Prothrombin time -INR	1.23 (1.02–2.03)	1.19 (1.08–1.38)	1.24 (1.02–2.03)	0.1059
Indocyanine green (%)	38 (2–78)	25 (2–56)	41 (22–78)	0.0024

Data are number of patients, except those denoted by ^a^, which represent the median (range) values

*INR* international normalized ratio

All patients underwent plain and contrast-enhanced computed tomography (CECT) examination with a multidetector CT scanner (Aquilion-16 or Aquilion-64; Toshiba Medical Systems, Tokyo, Japan), set at 5.0-mm slice thickness at 35, 60, and 180 s to obtain hepatic arterial, portal venous, and equilibrium phase images after the injection of contrast medium (1.5 mL/kg bodyweight; Iomeron™ 350 mg I/mL; Eisai, Tokyo) at a rate of 3.0 mL/s. Other parameters of the abdominal CT scan included tube voltage of 120 kVp, tube current of 240 mA, rotation time of 0.6 s, helical pitch of 1.375, field of view of 35–40 cm, and matrix of 512 × 512. Patients with iodine allergy underwent magnetic resonance imaging (MRI).

### Protocol for treatment of portal vein thrombosis

**Fig. 2:**
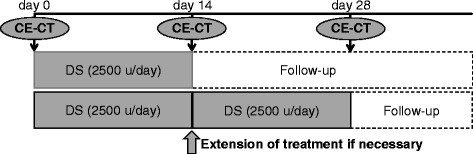
Protocol for monitoring and treatment of cirrhotic patients with PVT. Cirrhotic patients with PVT were treated with DS, 2500 units/day for 2 weeks. A second course of 2500 units/day for 2 weeks was administered in patients who showed no or partial response to the first course. CECT: contrast-enhanced computed tomography, DS: danaparoid sodium, PVT: portal vein thrombosis

The treatment protocol is shown in Fig. 2. Cirrhotic patients with PVT were treated with DS (Orgaran; MSD, Tokyo), 2500 units/day (IV drip) for 2 weeks. The same treatment was repeated for 2 weeks in patients who showed no or partial response to the initial course of treatment. All patients received warfarin as maintenance therapy. AT-III and other thrombolytic agents were not used during the administration of DS.

Esophageal and gastric varices were assessed endoscopically. Endoscopic injection sclerotherapy or endoscopic variceal ligation was used for treatment of varices assessed as F2 or F3 and/or RC1 or RC2/3 before anticoagulation therapy. Imaging studies, laboratory tests (hepatic reserve test, platelet count, and tests of the coagulation/fibrinolytic system) and complications were assessed before and after treatment.

### Evaluation of PVT

CECT was performed in all patients to determine the maximum extent of stenosis and presence of PVT. Multislice CT image data were reconstructed and transferred to a computer workstation (Ziostation; Ziosoft, Tokyo) for postprocessing. The response to treatment was categorized as complete response (CR, complete disappearance of thrombus), partial response (PR, ≥70% reduction in size of thrombus), and no change (no-change, <70% reduction in size of thrombus), compared with the pretreatment thrombus volume.

The study was approved by the institutional review board of the participating clinical sites, the Ethical Committee for Epidemiology of Toranomon Hospital, all protocols and amendments were approved by the ethics committee (#1096) and conform to the ethics guidelines of the 1975 Declaration of Helsinki. All participating patients provided written informed consent.

### Statistical analysis

The maximum extent of stenosis of PVT and differences in tumor response rate among the groups were analyzed by the chi-square test. Recurrence and survival rates were analyzed using the Kaplan-Meier technique with the log-rank test. A *P* value <0.05 was considered significant. Data were analyzed using IBM SPSS Statistics software (version 19).

## Results

### Effects of danaparoid sodium treatment on PVT

**Fig. 3:**
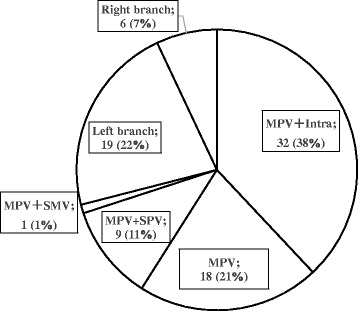
Sites of portal vein thrombosis. Thrombosis was located completely or partially within the main trunk of the portal vein in the majority of patients (71% of 90 patients)

Figure 3 illustrates the sites of PVT. Most thrombi (71% of 90 patients) were located completely or partially within the main trunk of the PV: in the proximal PV plus the intrahepatic branch in 32 (38%), in the proximal PV in 18 (21%), proximal PV plus splenic vein in 9 (11%) and proximal PV plus superior mesenteric vein in 1 (1%) patient.

Table 2 shows the response rates to DS after the initial course of 2-week treatment. The median values of the maximum extent of portal vein stenosis before and after treatment were 70% (range, 30–100%) and 20% (range 0–100%), respectively, and the median difference in the extent of stenosis before and after treatment was significant (*p* < 0.001). CR was noted in 44 (49%) patients, PR in 30 (33%), and NC in 16 (18%) patients.

**Table 2:** Therapeutic effects of the initial and repeat treatment with danaparoid sodium

Outcome	All patients (*n* = 90)	Acute type (*n* = 27)	Chronic type (*n* = 63)
Initial treatment			
Complete response	44 (49%)	23 (51%)	21 (33%)
Partial response	30 (33%)	2 (35%)	28 (45%)
No change	16 (18%)	2 (14%)	14 (22%)*
Repeat treatment	(n = 44)		
Complete response	10 (22%)		
Partial response	17 (39%)		
No change	17 (39%)		

*p = 0.001, compared with the complete and partial response groups, by chi-square test

Forty-four patients who developed recurrence required a repeat course of 2-week DS treatment. Table 2 shows the rates of response to DS after the second treatment. CR was obtained in 10 (22%), PR in 17 (39%), and NC in 17 (39%) patients. Retreatment was effective in 54% of the patients who required another course of DS treatment.

**Fig. 4:**
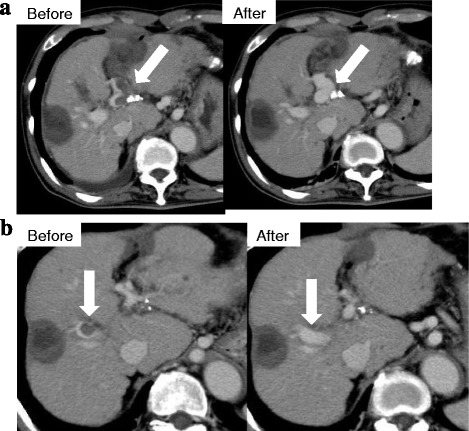
Representative case of complete response (CR) [Acute type]. **a** The patients was a 66-year-old man with type C cirrhosis, Child-Pugh B. PVT of the main branch of the portal vein disappeared after 2 weeks of treatment. **b** Recurrence in the right branch of the intrahepatic portal vein at 7 months after treatment. The patient was treated again for 2 weeks with DS. Maintenance therapy using warfarin maintained CR. *DS: danaparoid sodium, PVT: portal vein thrombosis, CR: complete response*

The radiological findings in a representative case of CR are presented in Fig. 4. This patient presented with PVT in the main branch of the PV, with maximum stenosis of 80% before and 0% after treatment. Recurrence was noted 7 months after the initial treatment with DS. DS was administered again for 2 weeks. This was followed by administration of warfarin as maintenance therapy. CR was maintained up to the time of writing of this report. No major or minor bleeding events, episodes of thrombocytopenia, or evidence of liver dysfunction were encountered during the 2 weeks of DS treatment.

### Changes in blood parameters

**Fig. 5:**
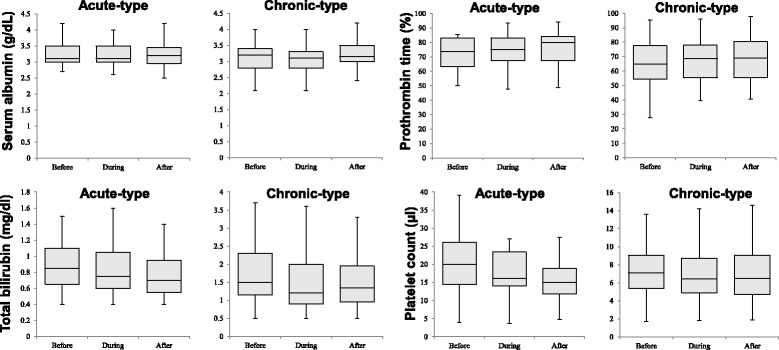
Effect of treatment with danaparoid sodium on serum albumin, total bilirubin, prothrombin time, and platelet count. No treatment-related significant changes were observed in these parameters. In these plots, lines within the boxes represent median values; the upper and lower lines of the boxes represent the 25th and 75th percentiles, respectively; and the upper and lower bars outside the boxes represent the 90th and 10th percentiles, respectively

Serum albumin, total bilirubin (T-Bil), prothrombin time (PT), platelet count did not change after therapy in both the acute and chronic categories (Fig. 5). These results indicated that DS does not affect liver residual function. AT-III activity at the start of therapy was significantly higher in patients who achieved CR or PR than in those of the NC group (*P* = 0.009). AT-III can be used for the treatment of patients with low AT-III activity**.** Serum D-dimer levels were significantly lower after DS therapy in both groups (*P* < 0.001).

### Cumulative PVT recurrence rate after the initial course of DS therapy

**Fig. 6:**
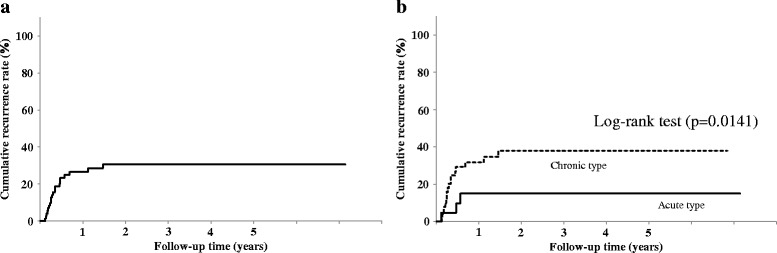
Cumulative recurrence rate of PVT. Recurrence rates at 1 and 3 years after treatment were 26 and 30%, respectively (**a**). Significantly fewer recurrences were noted in patients with Acute type than those with Chronic type (*P* = 0.0141) (**b**)

The recurrence rates at 1 and 3 years after treatment were 26 and 30%, respectively (Fig. 6a). Patients with Acute type had significantly fewer recurrences than patients with Chronic type (*P* = 0.0141) (Fig. 6b).

### PVT-related cumulative survival rate after initial DS treatment

**Fig. 7:**
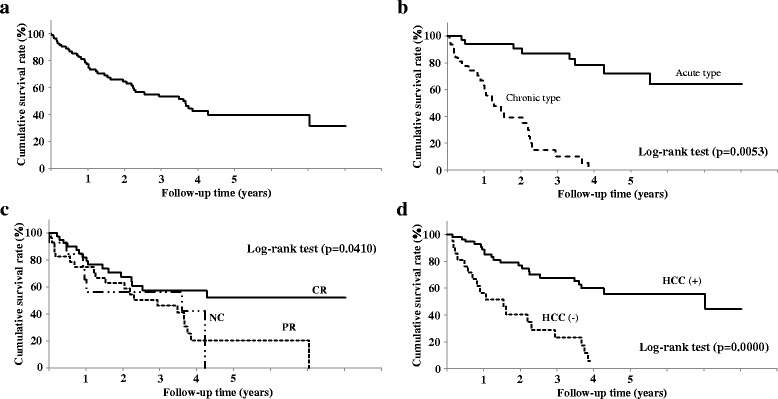
Cumulative survival rate of PVT. Cumulative survival rate for all patients (**a**), and according to the type of liver cirrhosis (**b**), response to treatment with danaparoid sodium (CR: complete response, PR: partial response, NC: no response) (**c**), and for patients with and without hepatocellular carcinoma (HCC) (**d**)

The cumulative survival rates at 1 and 3 years after the initial treatment were 83% and 60%, respectively (Fig. 7a). The survival rate was significantly higher in patients with acute cirrhosis than those with chronic cirrhosis (*P* = 0.0053) (Fig. 7b). The survival rate also varied according to the response to DS therapy and was higher in the order of CR, PR, and NC (P = 0.0053) (Fig. 7c). The cumulative survival rate was significantly higher for patients without HCC at initial treatment compared to patients with HCC (*P* = 0.0000) (Fig. 7d).

## Discussion

The results of this study showed that anticoagulation with DS is safe and effective treatment, significantly reducing the risk of progression of PVT and liver decompensation. In our study, the response rate (CR + PR) was higher than 75% in all the groups of patients tested in this study. These results are to a large extent similar to those reported by Naeshiro et al. [20], who reported response rate of 77% (CR 15%, PR 62%) after 2-week treatment with DS. Interestingly, the treatment was not associated with severe adverse events, such as gastrointestinal bleeding, thrombocytopenia, or worsening of liver dysfunction (Fig. 4).

Although no side effects were encountered, PVT relapse occurred in about third of the patients, though a second course of DS treatment was efficacious in 54% of the patients. In this regard, the second course of DS treatment was followed by warfarin for maintenance therapy. It is important to keep the international normalized ratio (INR) at less than 2 during warfarin use. The prognosis of patients with unresolved PVT remains poor, including death from hepatic failure.

The treatment goals in PVT include the reversal or prevention of progression of thrombosis in the portal venous system and prevention/treatment of complications. Management decisions must be tailored to the individual patient and should be based on the experience of the attending specialist, since there are only a few randomized controlled trials and no standardized treatment protocols are currently available. The current guidelines of the American Association for the Study of Liver Disease [9] recommend that all patients with acute PVT be anticoagulated for at least 3 months, starting with low-molecular-weight heparin (LMWH), followed by an oral anticoagulant. Long-term anticoagulation is recommended for patients with permanent risk factors or with distal extension of the thrombus into the mesenteric veins. In the setting of chronic PVT, patients should be screened for varices and receive appropriate prophylaxis, with long-term anticoagulation therapy being one consideration for patients with risk factors for thrombosis. At present, no standard recommendations exist for patients with cirrhosis.

DS is devoid of heparin or heparin fragments. Its antithrombotic activity has been well established. The exact antithrombotic mechanism of DS is unclear, but is thought to involve a complex interaction between its two major components. Laboratory monitoring is usually not necessary and bleeding enhancement by DS is minimal. However, patients with serum creatinine above 2 mg/dL should be monitored carefully. There is no antidote for DS, and protamine does not reverse its anticoagulant effect. DS is contraindicated in patients with severe hemorrhagic diathesis; active major bleeding; hypersensitivity to danaparoid, sulfates, or pork products; and those with positive in vitro test for antiplatelet antibodies in the presence of danaparoid.

Several studies have investigated the clinical value of warfarin (a vitamin K antagonist) in the treatment of PVT in cirrhotic patients. The rate of PV recanalization in warfarin-treated patients with cirrhosis is about 40% [21]. Orally administered warfarin is more acceptable to patients; however, treatment with warfarin is particularly difficult in patients with cirrhosis, primarily because monitoring of anticoagulation is complex in this particular situation. Notably, the results of assessments based on the INR in patients with liver disease often overestimate bleeding risk, because this index is determined in plasma samples from patients taking warfarin [22]. The INR has only been validated in individuals with normal liver function on stable anticoagulation. In this regard, 29% variation in mean INR values was reported in a study of patients with cirrhosis treated with one of three different thromboplastin reagents [23]. Further studies are needed to determine whether the target INR value ranging between 2 and 3 is adequate in individuals with abnormal INR values before anticoagulation therapy.

Administration of AT-III to patients with cirrhosis might be efficacious in the prevention of PVT. Kawanaka et al. [24] demonstrated that low and decreasing AT-III activity was associated with the development of PVT in patients with cirrhosis who have undergone splenectomy, and that treatment with AT-III concentrate would probably prevent the development of PVT in these patients.

Previous studies showed that the rate of portal vein recanalization was significantly higher while the rate of thrombus progression was significantly lower in the anticoagulation group compared with the non-anticoagulation group. These results suggest that anticoagulation, rather than “wait-and-see” strategy, should be actively employed to maximize the recanalization of thrombosed portal veins in liver cirrhosis. However, this recommendation should be cautiously applied for the following reasons. First, only a small number of studies were included in the two comparative analyses, and none of them was nonrandomized. Second, the role of spontaneous portal vein recanalization should be considered always [9, 21]. To avoid overtreatment, future studies of cirrhotic patients with PVT who benefit most from anticoagulation are warranted.

The timing and duration of follow-up are also controversial. We tend to see patients either in the surgical or specialized coagulation outpatient clinics every 3 months for at least 1 year. Depending on the location and the extent of thrombosis, CECT should be used regularly to assess the vessel patency.

The strength of our study relative to previous studies is that we used DS for 4 weeks rather than for only 2 weeks. However, our study has three important limitations. First, we used DS to treat PVT in patients with >70% stenosis. Treatment adaptation depends on the institution. Second, the study included only a small number of patients who received warfarin as maintenance therapy. We hesitate to use warfarin in patients with cirrhosis. Many novel oral anticoagulants have been approved for such patients, but since warfarin is an antagonist, it can be used relatively safely. Third, we did not examine the long-term outcome of patients after treatment and identified the factors that affect survival.

## Conclusions

Warfarin following danaparoid sodium for the treatment of PVT in patients with liver cirrhosis was safe and effective. An early diagnosis of PVT along with the evaluation of the volume of PVT on CT and an early intervention would contribute to the higher efficacy of the treatment. Thus, we recommend anticoagulation for the management of PVT in liver cirrhosis. Prevention of PVT or successful recanalization of a previously thrombosed portal vein can potentially improve survival of such patients.

## Abbreviations

AT: Antithrombin
CECT: Contrast-enhanced computed tomography
DS: Danaparoid sodium
HCC: Hepatocellular carcinoma (HCC)
LMWHs: Low-molecular-weight heparins
MRI: Magnetic resonance imaging
PVT: Portal vein thrombosis
